# Nocardial epidural abscess: A case report

**DOI:** 10.1016/j.inpm.2024.100395

**Published:** 2024-03-01

**Authors:** Michael Galibov, Michael Chung, Faraz Jamal, Aarsh Shah, Jeremy Benhamroun-Zbili, Mohamed Hasham, Alexander Shustorovich

**Affiliations:** a FK Johnson Rehabilitation Institute, Edison, NJ, USA; b Touro College of Osteopathic Medicine, Middletown, NY, USA; c JFK Hospital, Edison, NJ, USA

**Keywords:** Nocardia, Epidural abscess, Epidural steroid injections, Case report

## Abstract

Few cases of primary Nocardial epidural abscesses have been reported in the literature over the past 50 years, with limited guidelines available for identification and management. Typically, cases involve a prior diagnosis of systemic Nocardiosis with resultant seeding of a disseminated infection to the spine. An adult with chronic low back pain and type 2 diabetes mellitus underwent three consecutive epidural steroid injections in an outpatient setting. The patient gradually developed diffuse bilateral lower extremity pain, acute urinary retention, and saddle paresthesia. Lumbar magnetic resonance imaging revealed central herniation with annular tear compressing the thecal sac and S1 nerve roots, a dorsal epidural hemorrhage, and an abscess causing severe canal stenosis at L4-L5 and L5-S1. The patient was treated with vancomycin, piperacillin-tazobactam, and methylprednisolone without improvement, ultimately requiring surgical decompression. Initial surgical cultures grew mycobacterium species prompting RIPE therapy. Symptoms continually worsened requiring repeat decompression. Final cultures grew *Nocardia,* which necessitated transition to linezolid and sulfamethoxazole/trimethoprim, resulting in clinical improvement. Nocardial infection is a rare cause of isolated epidural abscess that can complicate antibiotic selection, resulting in potentially delayed treatment and worsened clinical outcomes. This manuscript aims to elucidate this rare but essential caveat to epidural abscess management.

## 1 Introduction

The Nocardia species are an obligate aerobe, gram-positive, branching filamentous, and partially acid-fast bacilli first described by Edmond Nocard in 1888 [1]. Nocardia species stem from soil, rotting plants, and other detritus [9]. Nocardial infection is often overlooked as symptoms may mimic other common sources of infection. Nocardia strains can take upwards of 21 days to appear on gram stains and do not result as positive on initial blood cultures. Nocardial infection, dubbed Nocardiosis, is rare, with a yearly incidence between 500 and 1000 cases in the United States [1,6]. Nocardia is predominantly an opportunistic bacterium targeting immunocompromised hosts [2]. The nidus of infection is most commonly attributed to the cutaneous, pulmonary, and central nervous systems (CNS) [1,5]. Pulmonary nocardiosis is the most common and disseminates widely. The CNS is ultimately involved in 44% of cases, with a mortality rate as high as 55% [2,7]. Dissemination to the CNS involves formation of multifocal brain abscesses with occasional presentations of meningitis, vertebral osteomyelitis, and spinal epidural abscesses [2,3]. Less than a dozen cases of primary Nocardial epidural abscesses have been reported in the literature over the past 50 years, with most cases precipitated by a previous diagnosis of systemic Nocardiosis [[2], [3], [4]]. We present a case of a patient who developed a Nocardial epidural abscess after receiving epidural steroid injections, without a prior history of systemic Nocardiosis.

## 2 Case report

**Fig. 1:**
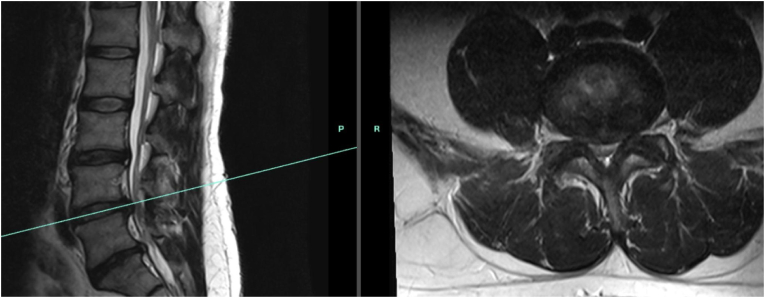
Outpatient MRI lumbar spine without contrast prior to hospital presentation.

A 39-year-old male with a past medical history of chronic low back pain, type 2 diabetes mellitus (A1C of 10.7), alcoholism in remission, and bipolar disorder presented to the pain clinic with bilateral leg pain and heaviness. The patient sought medical attention after suffering an acute flare of back pain with radicular symptoms while exiting their car. They were evaluated by an outside physician and reportedly found to have multiple herniated discs, uncertain of the specific levels involved. The patient subsequently underwent a lumbar epidural steroid injection (ESI) at a private pain management clinic. Details regarding the use or type of image guidance remained unclear as per the patient. However, during the procedure, the patient reported severe “burning, killing pain from head to toe” associated with a positional headache. Patient noted seeing a parrot in the waiting room of the clinic, separated from the procedural area by a door that frequently opens. One week later, the patient presented to our pain clinic complaining of bilateral leg fatigue, weakness, and dysesthesias which had been progressively worsening since the injection. Physical findings included hyperesthesia and allodynia of the right posterior calf, 4/5 strength with bilateral foot inversion and 3+/5 strength with right plantarflexion. The patient was referred for neurosurgical evaluation and nerve conduction study, as well as lumbar magnetic resonance imaging (MRI) without contrast which revealed multilevel central disc herniations with annular tears most severe at L4-5 and L5-S1 with compression of the thecal sac, right L4 nerve root, and S1 nerve roots (Fig. 1). The patient developed acute urinary retention, saddle anesthesia, and worsening bilateral lower extremity weakness prior to scheduled follow-up.

**Fig. 2:**
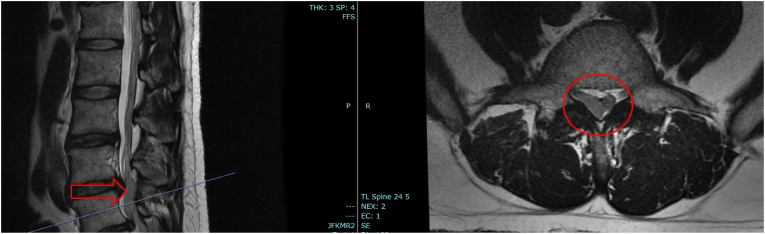
Mri lumbar spine without contrast demonstrating abscess at L4 - L5 level with extension caudally into the sacrum.

The patient was instructed to present to the emergency department where repeat lumbar MRI without contrast demonstrated lobulated dorsal epidural T2 hyperintense signal extending from the L4-L5 level into the sacrum, with concomitant severe canal stenosis at L4-L5 and L5-S1 (Fig. 2). Empiric vancomycin and piperacillin/tazobactam, as well as methylprednisolone were initiated, and the patient underwent emergent bilateral L4-L5 and L5-S1 micro-endoscopic decompression with epidural abscess evacuation and bilateral L5 laminectomy.

Postoperatively, the patient had improvement of urinary retention, saddle anesthesia, and lower extremity sensation and strength. Intraoperative cultures taken from the abscess fluid initially grew Mycobacterium, at which time the antimicrobial regimen was transitioned to rifamycin, isoniazid, pyrazinamide, and ethambutol (RIPE). The patient underwent tuberculosis screening and human immunodeficiency virus (HIV) testing which were negative, and final cultures grew *Nocardia nova*. Antimicrobials were then switched to linezolid and sulfamethoxazole/trimethoprim. During this transition, the patient traveled to India to visit family where they experienced worsening of their low back and leg pain and subsequently sought medical attention. Lumbar MRI was again obtained which revealed abscess formation at prior right L5 laminectomy site extending into the epidural space with evidence of arachnoiditis. The patient then underwent L5-S1 re-exploration with debridement of granulation tissue and lavage. Intraoperative cultures were initially negative but redemonstrated *Nocardia nova*.

On subsequent follow-up with our pain clinic, the patient noted an improvement in their back pain and radicular pain. Distal bilateral lower extremity numbness slowly improved, with continued burning sensation of the right heel managed with capsaicin patches. On exam, pin-prick sensation was diminished in the L4-L5 dermatomal distribution of the right lower extremity and absent in the S1 dermatomal distribution. Their bowel and bladder function returned to normal. The patient was compliant and tolerant of their antibiotic regimen consisting of linezolid and sulfamethoxazole/trimethoprim, with a plan to complete a 6–12 month course.

## 3 Discussion

In the United States, Nocardia infection is rare with a yearly incidence between 500 and 1000 cases [1]. Typically, Nocardiosis presents as a pulmonary infection with systemic symptoms such as fever, weight loss, and night sweats with the potential to spread to the CNS after pulmonary infection [6]. Primary CNS nocardial cases are rare, and very few have been reported in the literature in the past 50 years [2,7]. Our patient underwent lumbar epidural steroid injections for an acute flare of chronic low back pain and radiculopathy. The sterility of the procedure was questionable, as well as the report of the patient seeing a parrot in the office prior to the procedure. The patient also could not definitively recall whether the injections were performed under image guidance or with sterile technique. Moreover, the repetition of multiple epidural steroid injections and history of poorly controlled diabetes may have contributed to an immunocompromised state for this patient.

There were several challenges in the diagnosis and treatment of this patient. After the most recent epidural steroid injection, the patient developed cauda equina syndrome with imaging suggestive of epidural abscess. This collection is presumed to have formed over the course of 7–10 days as it was not clearly visualized on the initial lumbar MRI without contrast. Due to the delay in speciation and identification of the *Nocardia nova* pathogen, the patient was initially treated empirically for *Mycobacterium tuberculosis* [4]. The pathology of the Nocardial infection is comparable to other granulomatous infections and malignancies, and definitive diagnosis requires invasive sampling and growth on culture [2,7]. Once antibiotics were transitioned to linezolid and sulfamethoxazole/trimethoprim, specific to the sensitivities of *Nocardia nova*, the patient had significant improvement in pain and neurological impairment [6]. This case highlights that spinal epidural nocardiosis remains a rare occurrence within the United States with limited provider awareness, evidence, and resources in literature to guide identification and management [2]. Imaging with contrast might have contributed to earlier identification of fluid collection. Additionally, there are many obstacles in identifying and treating Nocardial epidural abscesses, including limited guidelines for testing and culturing, slow progression of Nocardial infections in culture, and the general non-specific clinical presentations of nocardiosis**,** making it indistinguishable from other pathogens causing epidural abscesses [1,3,7]. Most cases of iatrogenic nocardial abscesses result from poor sterile technique. Therefore, clinicians must always implement sterile techniques for neuro-axial procedures. If an infection is suspected, providers must remain vigilant if “Mycobacterium” is seen on culture, as this case demonstrates Tuberculosis may not be the default culprit [5,8].

## Disclaimer

Patient has signed a release for review of this case to medical journals with the understanding that he will not be receiving any compensation. The purpose of this report is purely for academic sharing of information and no authors have any conflicts of interest or vested interests involved in this case. No grants or outside funding were utilized for this case report. This manuscript has not been published in a prior form and is not pending at other journals at this time.

## Declaration of competing interest

The authors declare that they have no known competing financial interests or personal relationships that could have appeared to influence the work reported in this paper.
